# CAR T-Cell Therapy in Hematological Malignancies

**DOI:** 10.3390/ijms22168996

**Published:** 2021-08-20

**Authors:** Theresa Haslauer, Richard Greil, Nadja Zaborsky, Roland Geisberger

**Affiliations:** 1 Department of Internal Medicine III with Haematology, Medical Oncology, Haemostaseology, Infectiology and Rheumatology, Oncologic Center, Salzburg Cancer Research Institute-Laboratory for Immunological and Molecular Cancer Research (SCRI-LIMCR), Cancer Cluster Salzburg, Paracelsus Medical University, 5020 Salzburg, Austria; Th.haslauer@salk.at (T.H.); r.greil@salk.at (R.G.); n.zaborsky@salk.at (N.Z.); 2 Department of Biosciences, Paris Lodron University of Salzburg, 5020 Salzburg, Austria

**Keywords:** CAR T-cells, hematological malignancies, leukemia, lymphoma

## Abstract

Chimeric antigen receptor (CAR) T-cells (CAR T-cells) are a promising therapeutic approach in treating hematological malignancies. CAR T-cells represent engineered autologous T-cells, expressing a synthetic CAR, targeting tumor-associated antigens (TAAs) independent of major histocompatibility complex (MHC) presentation. The most common target is CD19 on B-cells, predominantly used for the treatment of lymphoma and acute lymphocytic leukemia (ALL), leading to approval of five different CAR T-cell therapies for clinical application. Despite encouraging clinical results, treatment of other hematological malignancies such as acute myeloid leukemia (AML) remains difficult. In this review, we focus especially on CAR T-cell application in different hematological malignancies as well as strategies for overcoming CAR T-cell dysfunction and increasing their efficacy.

## 1. Introduction

In cell-mediated immune responses, T-lymphocytes (T-cells) play a pivotal role in surveilling and eliminating tumor cells or pre-malignant cells. If T-cell activity is impeded, cancer can develop [1]. Since many cancer types acquire the ability to silence anti-cancer immune responses, scientists have developed strategies to fight back with immunotherapy, based on boosting a patient’s own immune system to attack the cancer cells [2]. T-cell-based adoptive immunotherapy is an approach to modify and redirect T-cells against cancer cells. As a part of this, CAR T-cell therapy is a relatively new treatment option, based on reprogramming a patient’s own T-cells with a CAR construct and returning them into the patient’s blood, where they start to attack cancer cells [3]. This technique was first demonstrated by the Eshhar lab, which paved the way for a chimeric cancer therapy [4]. The CAR itself functionally replaces the endogenous T-cell receptor (TCR) and is a hybrid protein composed of four different components. The extracellular domain is usually a single-chain variable fragment (scFv) derived from a Fab or a monoclonal antibody coupled via a flexible linker determining the antigen specificity. The hinge region derived from CD4 or IgG4 connects the extracellular- to the transmembrane domain and is important for conformational flexibility. The intracellular domain is composed of a co-stimulatory domain like CD28, 4-1BB, ICOS or OX40 imitating the costimulatory signal of the TCR during activation. The stimulatory domain represents the CD3ζ chain of a TCR or FcRγ finalizing the activation process [5,6,7]. The activated CAR T-cells specifically identify targets on cancer cells leading to their destruction. A main advantage herein is that the recognition is unrestricted to the MHC. The first application field of CAR T-cell therapy has been hematological malignancies like ALL, chronic lymphocytic leukemia (CLL) and multiple myeloma (MM) since they are easier to target than solid cancers in regard to finding an adequate tumor antigen [8,9]. So far, five CAR T-cell therapies have been approved by the food and drug administration (FDA), four of them targeting CD19, the most frequently used antigen. Recently, in March this year, an anti-BCMA CAR T-cell therapy (Idecabtagene viclaucel) for the treatment of multiple myeloma has been approved [10]. However, various hematological diseases such as acute myeloid leukemia (AML) or Richter’s syndrome still lack successful breakthroughs in CAR T-cell therapy for treatment of those diseases [11]. In this review, we want to provide an updated overview of CAR T-cell treatment options in hematological malignancies as well as address strategies to overcome CAR T-cell dysfunction and new approaches for combination with other therapies, which will undoubtedly change the field of autologous T-cell immunotherapy.

## 2. CAR T-Cell Therapy in Hematologic Malignant Neoplasms

**Figure 1:**
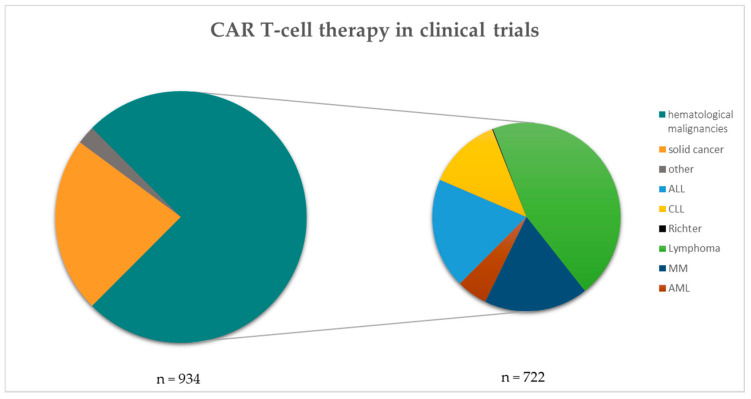
CAR T-cell therapy in clinical trials. The left pie chart shows the number (n) of CAR T-cell therapies in clinical trials categorized into solid cancers, others and hematological malignancies (n = 934). The hematological malignancies are further listed within the right pie chart (n = 722). Data taken from clinicaltrials.gov and filtered for each disease separately [13]. Search criterion was “CAR” and all hits were manually filtered for each category shown.

Until today, CAR T-cell therapy is mainly performed in the context of hematological malignancies, but an increasing number of trials are also conducted in solid tumor patients (Figure 1; clinicaltrials.gov) [12]. In this section, we focus on CAR T-cell therapy in leukemias, lymphomas and myelomas.

### 2.1. CAR T-Cell Therapy in Acute Lymphoblastic Leukemia

ALL is caused by malignant precursor B- or T-lymphocytes affecting normal blood cell production in the bone marrow [14]. It is the most common form of leukemia in children with a better prognosis compared to adults [15]. The incidence of B-ALL in adults is much higher compared to T-ALL [16]. Frontline therapy is usually chemotherapeutics. In high-risk patients, classified based on immunophenotype, somatic genetic alterations, site of relapse, prior therapy and time until relapse, an allogeneic hematopoietic stem cell transplantation in first remission as well as targeted immunotherapy is additionally advised [17,18]. To date, one CAR T-cell therapy is approved by the FDA, namely Kymriah [19] by Novartis, demonstrating marked effects in treating B-ALL, with 81% overall remission within 3 months [20]. Kymriah targets CD19, a B-cell surface marker, leading to a well tolerable B-cell aplasia as an off-tumor effect [21]. Since then several other trials started using different CAR constructs, stimulatory and co-stimulatory domains and adjusted manufacturing processes, because some patients still become insensitive to CAR T-cell therapy due to antigen loss of the tumor or CAR T-cell exhaustion [22]. Therefore, other targets instead of CD19 are being used. Currently more than a hundred clinical studies are registered investigating multiple targets, varying from CD20 as a potential target to bispecific CAR T-cells using CD19 and B-cell maturation antigen (BCMA) [23]. The AMELIA study using CD19 and CD22 as target achieved over 75% complete responders in three different groups varying in the administered dose of CAR T-cells (NCT03289455). To treat T-ALL, CAR T-cell therapies in clinical trials are targeting CD7 (NCT04572308, NCT04033302) or CD5 (NCT04594135) for example.

### 2.2. CAR T-Cell Therapy in Chronic Lymphocytic Leukemia

CLL is a very heterogeneous disease characterized by the accumulation of CD5/CD19 double-positive B cells in peripheral blood and lymphoid compartments. CLL is accompanied by immune dysregulation such as T-cell abnormalities including impaired synapse formation, impaired proliferative capacity of T-cells, exhausted T-cell phenotype and a diminished ability for T-cells to execute cytotoxicity [24]. The risk of suffering from the disease increases with age and is more common in the western world [24,25]. The conventional therapy for symptomatic CLL patients includes monoclonal antibodies, chemotherapy and immunotherapy depending on diagnosis and progression of the disease [25,26]. Although there are already a plethora of new therapeutics including BTK inhibitors, PI3K inhibitors, BCL2 inhibitors and Fc-engineered monoclonal antibodies for example, CLL is still mostly incurable [25]. CAR T-cell therapy has been investigated for patients with relapsed or refractory disease using mostly CD19 as a target. Compared to ALL or diffuse large B-cell lymphoma (DLBLC), response rates are by far worse in CLL. In a study by Geyer et al., the overall response rate was only 38% and the complete response rate was 25% with a median overall survival of 17 month [27]. Frey et al. investigated an overall response rate of 44% with only 28% of complete responders. The median overall survival was 64 month in this study [28]. Despite the challenges and relatively low response rates in CLL, there are potential applications for CAR T-cell therapy in CLL. Some clinical studies are focusing on CAR T as a consolidation therapy for patients with incomplete remissions [29]. Furthermore, this could be a potential application in elderly patients with comorbidities as a therapy with less adverse events compared to an allogeneic transplantation.

#### CAR T-Cell Therapy in RICHTER’S Syndrome

Richter’s syndrome is usually the transformation of a CLL into a higher malignant form such as a diffuse large B-cell lymphoma with a relatively poor prognosis. The disease is very aggressive and the median survival is five to eight months [30]. Since many patients with Richter’s syndrome have undergone extensive treatment before the transformation of the disease, treatment options are limited. In younger patients, an allogeneic hematopoietic stem cell transplant (HSCT) is indicated, in adult patients an immunotherapy is indicated [31]. Although the CAR T-cell therapy was firstly examined in CLL, it may help also Richter’s syndrome patients with limited treatment options [30,31]. The Mayo clinic in Rochester, Minnesota, started a clinical trial very recently in May this year enrolling patients with relapsed/refractory B cell malignancies including Richter’s syndrome to be treated with CD19 directed CAR T-cell therapy (NCT04892277). Kittai et al. reported in their study at the Ohio State University James Comprehensive Cancer Center about nine patients receiving the CD19-directed CAR T-cell therapy axicabtagen-ciloleucel [32]. Eight of the nine patients were pretreated with kinase inhibitors and one patient died due to an infection. Five of these eight patients showed a complete response and three a partial response. So far, only one patient relapsed. Despite these encouraging results, far more investigation in this field is needed.

### 2.3. CAR T-Cell Therapy in Lymphoma

The first approved CAR T-cell therapy was the CD19-directed Kymriah for treating relapsed and refractory ALL and diffuse large B cell lymphoma (DLBCL) [19]. DLBCL is one of the most common forms of non-hodgkin lymphomas (NHL) and make up to 40% of all lymphomas [33]. In the ZUMA study, patients with refractory large B-cell lymphomas were treated with CD19 targeted CAR T-cells (Yescarta) showing 58% complete responders and 25% partial responders [34]. Durable responses of over two years could be seen, leading to the FDA approval of Yescarta (axicabtagene ciloleucel) in 2017 [35,36]. Recently in March 2021, a new CAR T-cell therapy was approved by the FDA, namely Breyanzi (Lisocabtagene marleucel) for treating refractory large B-cell lymphomas, such as DLBCL, high grade B-cell lymphoma, primary mediastinal large B-cell lymphoma and follicular lymphoma [37]. For the treatment of mantle cell lymphoma (MCL), the FDA approved the anti-CD19 CAR T-cell therapy Tecartus (Brexucabtagene autoleucel) [38]. So far, only CD19 targeted therapies for B cell lymphoma are approved indicating a need for changing the focus also to other targets. A study from 2014 (NCT01735604) revealed a response in 4 out of 7 patients treated with CD20 CAR T-cells [39]. Another potential target is CD30, a membrane protein on activated B-and T-cells belonging to the TNF receptor family. In a study treating Hodgkin lymphoma (HL) patients with CD30 CAR T-cells, seven out of 18 patients achieved a partial response [40]. Further investigation will be necessary to unravel new targets making CAR T-cell therapy applicable for a wide variety of patient’s characteristics [41].

### 2.4. CAR T-Cell Therapy in Multiple Myeloma

In multiple myeloma, malignant plasma cells accumulate in the bone marrow repressing normal hematopoietic cell production and further repressing osteoblast function [42]. This leads to the production of complete and incomplete immunoglobulins, so called paraproteins with no function. To date, the disease is almost incurable and various therapies, including chemotherapy, HSCT and immunomodulatory drugs, can only keep the disease stable over time and relieve symptoms [43]. CD19 targeted CAR T-cell therapies seem to be incapable of curing MM and achieve only minor effects in MM patients since CD19 is only expressed in low amounts on their surface [44]. Since then, several clinical trials are investigating different targets, above all, BCMA, which is expressed on mature B-cells and plasma cells, making it a promising target for CAR T-cell therapy in MM [42]. In a phase I CRB-402 clinical trial, CAR T-cells targeting BCMA (NCT03274219) were tested, showing a response rate of 86%. Further studies are investigating new targets and currently over hundred are registered for treating MM with CAR T-cell therapy [13]. CD138 or Syndecan-1 is especially expressed on MM cells and is therefore an interesting new target. A small clinical study (NCT01886976) assessed the safety and efficacy of a CD138 directed CAR T-cell therapy and explored a response rate of 80% showing a stable disease for over three months [45]. Recently, the first anti-BCMA CAR T-cell therapy named Abecma (Idecabtagene vicleucel) has been approved by the FDA for the treatment of relapsed and refractory MM.

### 2.5. CAR T-Cell Therapy in Acute Myeloid Leukemia

AML is a disease of the myeloid blood cell lineage arising mostly from genetic or epigenetic changes affecting normal blood cell production in the bone marrow [46]. Besides chemotherapy, an allogeneic hematopoietic stem cell transplantation can help to induce complete remission. Since AML is a genetically heterogeneous disease, characterizing the disease determines therapy options [47]. The major limitation for the usage of a CAR T-cell therapy in AML is the absence of a targetable antigen since many myeloid antigens are also expressed on healthy hematopoietic stem and progenitor cells (HSPCs) leading to destruction of the bone marrow [11]. As a consequence, targets have to be chosen carefully while achieving only minor and tolerable toxicities for the patients. The first AML CAR T-cell therapy was directed against the Lewis Y antigen showing only very limited efficacy [48]. By now, over twenty clinical trials are enrolling and recruiting patients for CAR T-cell therapy in AML targeting predominantly CD123, CD33 and CLL-1. CD123 and CD33 are mainly expressed on AML blasts; however, they can also be found on healthy HSCPs [49]. CLL-1 is highly expressed in AML but also on monocytes and other non-hematological cells [50]. Since the response rates are limited so far, scientists go for combinatory targets in CAR T-cell therapy. For example, patients are currently being recruited at Zhujiang hospital in China for treatment with CD38/CD33/CD56/CD123/CD117/CD133/CD34/Mucl-CAR T-cells (NCT03473457). A clinical trial at the Dana-Farber Cancer Institute in Boston, Massachusetts, used CAR T-cells targeting NKG2D-ligands (NCT02203825), which showed very poor responses in acute myeloid leukemia/myelodysplastic syndrome or relapsed/refractory multiple myeloma, with all patients receiving follow-up alternative therapies [51]. Further clinical trials target other antigens such as CD44v6 (NCT04097301), which are currently recruiting patients.

## 3. Overcoming CAR T-Cell Dysfunction

Antigen recognition is a crucial point in CAR T-cell therapy since many patients experience a relapse because the tumor cells become negative to the target antigen. Conversely, off-target cross reactivity in CAR T-cell therapy is still a problem. Hence, a major challenge is to improve the antigen recognition and specificity of CAR T-cells. Bispecific CAR T-cells recognize two or more tumor associated antigens simultaneously, for example, CD19 and CD20 [52]. Furthermore, mixing different CAR T-cells that target the same antigen or tandem CARs (TanCAR) co-targeting two different tumor antigens may enhance therapeutic efficacy [53]. Enhancing proliferative capacity and persistence of CAR T-cells can be addressed via optimizing costimulatory signaling domains. Incorporating one or more costimulatory domains into the CAR construct can influence their effector function. CD28 and 4-1BB are widely used, but ICOS, OX40, CD27 and many more are also under investigation [54,55,56,57,58]. CAR T-cells based on 4-1BB costimulation are known to have a greater persistence while CD28 costimulation enhances proliferation and tumor elimination [59]. Another strategy is to modify cytokine expression via so-called T-cells redirected for universal cytokine killing (TRUCKS). Those 4th generation CAR T-cells deliver a transgenic protein of interest to the targeted tissue upon antigen encountered signaling. In detail, those CAR T-cells are synthetically engineered to express an inducible expression cassette driven by a transcription factor, leading to the expression of the transgenic cytokine upon signaling [60]. Furthermore, Shum et al. created transgenic T-cells with IL-7 receptors (C7R) incorporated in the CAR construct. Constitutive signaling is promoted when encountering an antigen, thus activating intracellular STAT5 signaling, the major IL-7 signaling nodal point, supporting anti-tumor activity [61]. Optimization of structural components can also include knocking out negative regulators, which is a powerful tool to overcome an immunosuppressive tumor microenvironment (TME). Immune checkpoint inhibitors play pivotal roles in tumors—the T-cell interactions lead to T-cell exhaustion, tolerance and ultimately dysfunction [62]. The CRISPR/Cas9 tool enables one to knockout immune checkpoint molecules such as PD-1, CTLA-4 and LAG3 in CAR T-cells [63]. The knockout of negative regulators such as transcription factors, for example NR4A, correlating with PD-1 and TIM3 gene expression, can help to induce tumor regression [64]. Expression of a dominant negative receptor (DNR) on the surface of a CAR T-cell targets the same goal. Engineered PD-1 DNR lacks PD-1 transmembrane and intracellular signaling domains augmenting CAR T-cell cytotoxicity [65]. Another synthetic biology approach is chimeric switch receptors (CSRs), which convert negative into positive signals by reversing the suppression of inhibitory molecules [66]. Liang et al. engineered CD19-targeted CAR T-cells expressing a PD-1 CSR, treating patients with post CD19 CAR T-cell failure to suppress PD-1/PD-L1-mediated T-cell exhaustion. Three of six patients achieved a complete response [67]. To abrogate and limit the cytotoxicity of CAR T-cells, they can be engineered with safety switches, which can inactivate and eliminate the CAR T-cells drug. Safety switches include suicide genes such as caspase 9 (iCasp9) fused with a FK506 binding protein, incorporated into the CAR, leading to dimerization and ultimately apoptosis upon addition of a synthetic inducer of dimerization drug [68]. Moreover, limiting CAR T-cell long term persistence can also prevent toxic effects. This can be achieved by using therapeutic antibodies, which specifically recognize CAR T-cells, leading to their elimination. These are just examples of all of the available powerful tools to modify CAR T-cells. Increased development of synthetic biology interventions are needed to facilitate personalized medicine in the field of CAR T-cell therapy.

## 4. CAR T-Cell Therapy and Combination Therapies

For lymphoma and ALL, CAR T-cell therapy has shown remarkable results in treating patients, but for CLL for example, results are not as promising [27]. Therefore, several studies are investigating CAR T-cell therapy in combination with other therapies to maximize the therapeutic efficacy but preserving patient safety at the same time. A research focus lies also on CAR natural killer cells (NCT04887012, NCT04887012) and CAR natural killer T-cells (NCT03294954).

### 4.1. Monoclonal Antibodies

Monoclonal antibodies used for cancer treatment either target tumor-associated antigens to induce cytotoxicity or are used to block receptor–ligand interactions. In this regard, immune checkpoint inhibitors are antibodies that block the inhibitory T-cell receptor CTLA-4 (Ipilimumab) or PD-1 (Pembrolizumab), which leads to reactivation of silenced cancer-specific T-cells [69,70]. As CAR T-cells also express multiple inhibitory receptors, combining CAR T-cell therapy with checkpoint blockage could possibly prevent the exhaustion and silencing of CAR T-cells. Chong et al. described a successful increase in CAR T-cell efficacy after treating a refractory DLBCL patient with pembrolizumab [71]. Only a few clinical studies so far are combining CAR T-cell therapy with monoclonal antibodies for the treatment of hematological malignancies (NCT04381741, NCT04703686, NCT03310619) and for the treatment of solid cancers (NCT03179007, NCT02862028, NCT01454596). In light of these results, combinatory therapy of CAR T-cells and monoclonal antibodies will be of more importance to emerge new strategies in fighting against cancers.

### 4.2. Small Molecule Inhibitors

Drugs smaller than 500 Daltons targeting distinct molecule portions are considered as small molecule inhibitors. Due to their size they are able to pass through the cell membrane to act intracellularly, antagonizing different pathways correlated with cancer development [72]. Tyrosine and serine kinase inhibitors are most frequently used to treat cancer patients targeting tumor survival, growth and metastasis [73]. The most promising target is the mitogen-activated protein kinase (MAPK) pathway since it is involved in multiple cellular functions. MEK inhibitors as well as BRAF inhibitors have shown impressive results for the treatment of solid cancer [74]. A clinical trial combining CAR T-cells and a BRAF inhibitor revealed mixed results as tumor infiltrating lymphocytes (TILs) were inhibited showing that the complexity of targeting that pathway in combination with adoptive T-cell therapy remains to be elucidated [75]. Since the PI3K/Akt/mTOR signaling cascade is a major key player in regulating the cell cycle, researchers demonstrated that Akt inhibition ex vivo could enhance antitumor immunity in CAR T-cell therapy [76]. Concerning mTOR inhibition, Huye et al. created rapamycin resistant anti-CD19 CAR T-cells and found out that those had an increased antitumor activity in Burkitt’s lymphoma and ALL cell lines [77]. One unpublished clinical trial is currently enrolling CLL and DLBCL patients in the United States, Australia and Europe for a combinatory therapy of CAR T-cells with Ibrutinib (NCT03960840). Furthermore, Fraietta et al. found out that Ibrutinib therapy administered before and during CAR T-cell treatment in CLL patients could improve CAR T-cell expansion and downregulation of inhibitory receptors [78].

### 4.3. Oncolytic Viruses

Oncolytic viruses target and eliminate tumor cells without damaging healthy tissue in two different ways. Firstly, through a direct attack, in which the virus infects and enters the cells, leading to cell lysis. Secondly, through expression of viral antigens in infected cancer cells, which leads to their subsequent recognition and destruction by cytolytic T-cells [79]. This principle was studied in MM cells using adenovirus serotype 5, showing oncolysis in infected malignant cells, suggesting an application also in other hematological malignancies [80]. Nishio et al. designed an oncolytic adenovirus armed with the chemokine genes RANTES and IL-15 leading to CAR T-cell recruitment, prolonged persistence and enhanced survival in neuroblastoma cell lines [81]. This could be an interesting attempt at combining CAR T-cells with oncolytic viruses for the treatment of hematological malignancies but also for solid cancer, where one phase I trial is running using a binary oncolytic adenovirus and HER2-targeted CAR T-cells (NCT03740256) for the treatment of HER2 positive solid tumors.

### 4.4. Proinflammatory Cytokines

Cytokines can tremendously influence T-cell function such as expansion, persistence and effector activity. In addition to the engineered co-expression of cytokines in CAR T-cells discussed in Section 3, cytokines can be administered intravenously to patients. For example, interleukin 2 (IL-2) influences T-cell growth, expansion and cytotoxicity, and is approved by the FDA for the usage in cancer treatment [82]. Several clinical trials are testing the combination of CAR T-cell therapy with IL-2 (NCT00924326, NCT00019136, NCT04119024, NCT03098355), revealing enhanced persistence of CAR T-cells and durable remissions in vivo in different tumor entities such as lymphoma, ovarian cancer and melanoma [83]. However, IL-2 is a double-edged sword as high IL-2 dosages can decrease central memory T-cells [84]. Other investigated cytokines such as IL-7 and IL-15 showed increased CAR T-cell cytotoxicity compared to IL-2 in ALL/CLL patients [85]. Most clinical trials are comparing IL-2 and IL-7/IL-15 activity in lymphoma patients (NCT02652910, NCT04186520, NCT03929107, NCT02992834), revealing the demand for testing combinatory approaches of CAR T-cells and proinflammatory cytokines.

## 5. Adverse Events of CAR T-Cell Therapy

Toxic effects frequently accompany curative effects of a CAR T-cell therapy. The most frequent side effect is the cytokine release storm (CRS), where excessive release of cytokines is triggered by CAR T-cell activation, proliferation and enhanced killing, manifesting in a broad range of clinical symptoms such as fever, tachycardia and pyrexia or even death [86]. Tocilizumab, a monoclonal antibody against the IL-6 receptor, acting as an immunosuppressant is often used for the treatment of CRS [87,88]. Besides CRS, tumor lysis syndrome (TLS) is a common toxicity upon CAR T-cell treatment. Due to mass destruction of malignant cells, their cellular components are rapidly released, leading to hepato- and nephrotoxicity. Overlapping with CRS, TLS can also lead to cardiac arrhythmia. Management of TLS should therefore include prevention of cardiac dysrhythmias as well as prevention of renal function [89]. A prevalent side effect includes neurotoxicity, which is generally associated with CRS. As CAR T-cells also migrate into the cerebrospinal fluid, high levels of cytokines in the cerebrum can lead to aphasia, delirium, seizures and syncope for example. For the management of neurotoxicity, corticosteroids are favored as they can pass the blood–brain barrier [90]. Furthermore, on-target off-tumor effects frequently occur when the CAR-target antigen is not exclusively expressed on tumors but also on healthy tissue. For example, B-cell aplasia occurs as an on-target off-tumor effect since CD19 targeted CAR T-cells also eliminate CD19 positive B-cells. However, B-cell aplasia upon CAR T-cell therapy is usually well-tolerated [91].

## 6. Conclusions

Immunotherapy and especially CAR T-cell therapy has demonstrated outstanding response rates in subgroups of patients with hematological malignancies, leading to emergence of CAR T-cells as a major breakthrough in cancer immunotherapy. Furthermore, the fifth CAR T-cell therapy has been approved by the FDA (Breyanzi), underlining that CAR T-cells have become a valid therapy option for refractory blood cancer and points to the promising potential of this therapy approach. However, in some hematological malignancies, response rates are low and patients still relapse. Additionally, for some hematological malignancies such as Richter syndrome, data is still very thin with only a low number of patients enrolled in clinical studies so far. In addition, adverse events frequently accompany CAR T cell therapy, showing that this therapeutic approach still needs to be optimized in regard to safety and efficacy. However, so far, four out of the five FDA-approved CAR T cell drugs target CD19 (Breyanzi, Kymriah, Tecartus and Yescarta) and only one targets a different antigen (BCMA, Abecma). Comparing these drugs with the expanding list of targets currently investigated in many clinical studies gives confidence that the number of approved CAR T constructs as well as the list of targets are still growing. This is particularly important as suitable targets for some entities such as AML are still missing. Aside from the quest for novel targets, a large panel of innovative approaches are expected to markedly improve CAR T cell therapy, which have been discussed in this review and comprise the development of bispecific CAR T cells, improved CAR constructs, genetic modification of CAR T cells and combination treatments with other drugs. Regarding all these technical possibilities, it is expected that the next generation of CAR T cells will hopefully serve as a safe and highly effective weapon to fight hematological malignancies.

## Data Availability

Not applicable.

## Abbreviations

ALL	acute lymphocytic leukemia
AML	acute myeloid leukemia
BCMA	B-cell maturation antigen
CAR	chimeric antigen receptor
CLL	chronic lymphocytic leukemia
CRS	cytokine release storm
CSR	chimeric switch receptors
DLBCL	diffuse large B-cell lymphoma
DNR	dominant negative receptor
FDA	food and drug administration
HL	Hodgkin lymphoma
HSCT	hematopoietic stem cell transplant
HSPCs	hematopoietic stem and progenitor cells
MAPK	mitogen-activated protein kinase
MCL	mantle cell lymphoma
MHC	major histocompatibility complex
MM	multiple myeloma
NHL	Non-Hodgkin lymphomas
scFv	single-chain variable fragment
TAA	tumor-associated antigens
TanCAR	tandem CAR
T-cells	T-lymphocytes
TCR	T-cell receptor
TILs	tumor infiltrating lymphocytes
TME	tumor microenvironment
TRUCKS	T-cells redirected for universal cytokine killing
VEGF-A	vascular endothelial growth factor A
